# Virtual surgical myectomy as a planning tool for obstructive hypertrophic cardiomyopathy

**DOI:** 10.1186/1532-429X-18-S1-P46

**Published:** 2016-01-27

**Authors:** Prahlad G Menon, Parachuri V Rao, Srilakshmi M Adhyapak, Ou Yuanchang, Richard Weeks

**Affiliations:** 1 grid.255272.50000000123643111Biomedical Engineering, Duquesne University, Pittsburgh, PA USA; 2 grid.21925.3d0000000419369000Bioengineering, University of Pittsburgh, Pittsburgh, PA USA; 3 grid.416432.60000000417708558Cardiology, St. John's Medical College hospital, Bangalore, India; 4 grid.416504.2Heart failure and Transplantation Program, Narayana Hrudayalaya Institute of Medical Sciences, Bangalore, India; 5 grid.12981.33000000012360039XSchool of Information Science and Technology, Sun Yat-sen University, Guangzhou, China; 6 QuantMD LLC, Pittsburgh, PA USA

## Background

Surgical myectomy is regarded as a gold standard treatment for Hypertrophic Cardiomyopathy (HCM) and may be conducted using different approaches, depending on the location of the thickened heart muscle, to widen the outflow tract and thereby reduce outflow drag to favorably alter the natural course of HCM. We present a novel approach for patient-specific visualization of precise locations of left ventricular (LV) hypertrophy as well as virtual resection of obstructions to facilitate optimal surgical myectomy.

## Methods

The LV endocardium and epicardium were reconstructed in 3D from short-axis cine steady-state free precession (SSFP) cardiac magnetic resonance (CMR) images of five HCM patients. Pre-operative LV volumes were quantified and the location as well as extent of regional hypertrophy was visualized at end-diastole using color-maps of myocardial wall thickness. Next, virtual myectomy was performed to resect the LV in the end-diastolic phase, interactively using a pre-surgical planning software, QuantMD Surgery Explorer (QuantMD LLC, Pittsburgh, PA). Simulative of myectomy, a real-time, free-form surface deformation tool was used for endocardial point cloud morphing and surface fitting (by Poisson surface reconstruction) to eliminate obstructive hypertrophic muscle, at several surgeon-selected endocardial sites. The spatial influence of deformation at each site was dictated by a Gaussian function affecting normally-outward endocardial vertex movement, expressing spatial influence as a fraction of the Euclidean distance between any given endocardial vertex and a surgeon-specified site of myectomy and magnitude of myectomy at each site by the Gaussian's amplitude.

## Results

**Figure 1:**
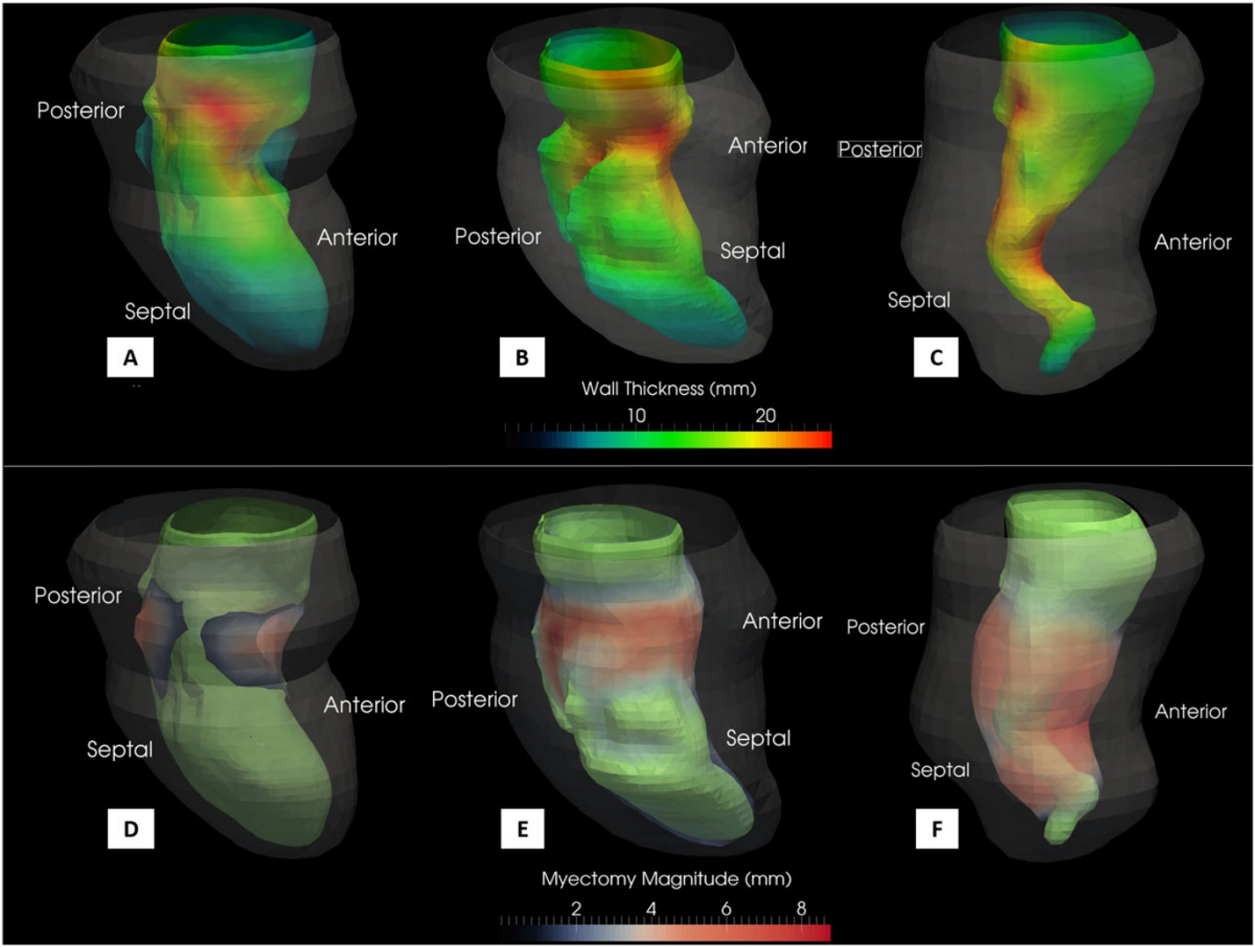
**3D renderings of the pre-operative (A,B,C) and virtual post-operative (D,E,F) LV endocardial surfaces of three representative patients in the study cohort**. In A, B and C, the regional wall thickness is color-mapped from blue to green to red indicative of increasing thickness (in mm units). In D, E and F, sites of myectomy are color-mapped from blue to red indicative of the extent of myectomy performed (in mm units) with the pre-operative endocardial shape shown as a green surface, for reference.

The study cohort (n = 5) had a mean LV mass of 197.3 ± 3.51 g, mean end-diastolic volume (EDV) of 60.8 ± 21.49 mL and mean stroke volume of 47.4 ± 15.27 mL. After virtual myectomy, the mean EDV increased significantly to 85.6 ± 12.78 mL (p = 0.057) with a significant increase in the stroke volume to 71.54 ± 14.68 mL (p = 0.034, end systolic volume is unchanged). Color-mapped endocardial surfaces were used to visualize the precise regional extent of virtual myectomy (in mm; see Figure 1D-1E) and retrospectively review results in the context of the pre-operative regional myocardial wall thickness (visualized separately in mm; see Figure 1A-1C) to judge completeness of the procedure.

## Conclusions

Virtual surgical modeling was employed to leverage patient-specific CMR imaging to guide myectomy in five hypertrophic cardiomyopathy patients presenting with myocardial obstructions in the LV outflow tract, mid-ventricular or apical regions. Virtual myectomy was conducted at the end-diastolic cardiac phase, simulative of the likely shape of the LV in the cardioplegic state. Our methodology offered realistic & dexterous control of virtual resection and may help avoid excessive resection or damage to adjacent ventricular structures while optimizing the precise location and extent of hypertrophic myocardial resection.

